# MULTIMORBIDITY PREDICTS REMAINING LIFESPAN IN NONHUMAN PRIMATE MODEL OF ACCELERATED AGING

**DOI:** 10.1093/geroni/igad104.2491

**Published:** 2023-12-21

**Authors:** Ellen Quillen, Jamie Justice, George Schaaf, John Olson, J Mark Cline

**Affiliations:** Wake Forest School of Medicine, Winston Salem, North Carolina, United States; Wake Forest University School of Medicine, Winston-Salem, North Carolina, United States; Wake Forest School of Medicine, Winston-Salem, North Carolina, United States; Wake Forest School of Medicine, Winston Salem, North Carolina, United States; Wake Forest School of Medicine, Winston Salem, North Carolina, United States

## Abstract

Multimorbidity is common among older adults and linked to shorter lifespans, but it is unclear how naturally-occurring multimorbidity impacts lifespan in non-human primate models of aging. We developed a clinical multimorbidity index based on standardized veterinary diagnoses in 288 rhesus macaques (Macaca mulatta) from the Wake Forest Radiation Late Effects Cohort, animals that survived an early-life insult from whole-body radiation and controls (73 female, 237 irradiated, ages 2-24). Our index is based on diagnosis of arthritis, low bone density, cataracts, diabetes, gastrointestinal disorders, heart disease, hepatic dysfunction, hypertension, kidney disease, lung fibrosis, skin disorders, tumors, and overweight/underweight. All diseases were seen in both control and irradiated animals and there was a broad distribution of multimorbidity trajectories across the lifespan both in number and combinations of diseases. On average, the first diagnosis occurred at age 9.0 (equal to 35% of median lifespan) and the second at age 10.7. However, 20% of animals that lived to age 12 were without any diagnoses while others had as many as 9 comorbidities, highlighting broad variability in multimorbidity with aging. Cox proportional-hazard ratios (HR) show significant negative associations (p < 0.05) between survival and multimorbidity. Ages 3-6 years, each additional morbidity doubles likelihood of death (HR > 2) with HR falling from 1.9 to 1.4 from ages 6-15. Based on receiver operating characteristic (ROC) curves, multimorbidity predicts 2-year mortality with AUC > 0.65 from ages 7-14. Our findings suggest multimorbidity is prevalent and predicts lifespan in NHP survivors of radiation. Research was funded by NIHU01AI150578.

